# Polydimethylsiloxane as a more biocompatible alternative to glass in optogenetics

**DOI:** 10.1038/s41598-023-43297-2

**Published:** 2023-09-26

**Authors:** Michael Aagaard Andersen, Jens Schouenborg

**Affiliations:** 1 Neuronano Research Center, Department of Experimental Medicine, Lund University, Lund, Sweden; 2 Department of Neuroscience, Faculty of Health and Medical Sciences, University of Copenhagen, Copenhagen, Denmark

**Keywords:** Neuroscience, Neural circuits, Neuronal physiology

## Abstract

Optogenetics is highly useful to stimulate or inhibit defined neuronal populations and is often used together with electrophysiological recordings. Due to poor penetration of light in tissue, there is a need for biocompatible wave guides. Glass wave guides are relatively stiff and known to cause glia reaction that likely influence the activity in the remaining neurons. We developed highly flexible micro wave guides for optogenetics that can be used in combination with long-lasting electrophysiological recordings. We designed and evaluated polydimethylsiloxane (PDMS) mono-fibers, which use the tissue as cladding, with a diameter of 71 ± 10 µm and 126 ± 5 µm. We showed that micro PDMS fibers transmitted 9–33 mW/mm^2^ light energy enough to activate channelrhodopsin. This was confirmed in acute extracellular recordings in vivo in which optogenetic stimulation through the PDMS fibers generated action potentials in rat hippocampus with a short onset latency. PDMS fibers had significantly less microglia and astrocytic activation in the zone nearest to the implant as compared to glass. There was no obvious difference in number of adjacent neurons between size matched wave guides. Micro PDMS wave guide demonstrates in vivo functionality and improved biocompatibility as compared to glass. This enables the delivery of light with less tissue damage.

## Introduction

Decoding the signals of specific neuron sub-populations with high temporal and spatial resolution in freely moving animals is essential for understanding the brain information processing that underlie complex motor behaviour, cognition, pain perception, mood, etc. Optogenetic tools offers the opportunities to manipulate activity in specific neuron populations during electrophysiological recordings and behavioural assessment^1–7^. For deep targets there is a need for biocompatible wave guides that leaves a minimal footprint on the tissue and the neuronal signalling. Flexibility, density (specific weight), and tethered attachment are all identified as important factors for biocompatibility and each individual factor correlates to the extent of glia-scar formation^8–13^. Current wave guides in glass are relatively stiff and may therefore produce substantial tissue reactions, including activation of microglia and astrocytes, implant encapsulation and altered neuronal activity^14–21^. Commercially available wave guides are made from two layers of glass and have a high stiffness and substantially higher density than the brain. An alternative material might be polydimethylsiloxane (PDMS), due to its low specific weight (1.03 g/cm^3^, product data sheet of Sylgard 184, Dow Chemical Company. Specific weight of brain tissue; 0.990–0.994 g/cm^3^)^22^ that is very close to that of brain tissues (thereby minimizing relative moment of inertia between tissues and implant^11^), high flexibility, tunable refractory index and easy handling^23–29^ (for reviews^30,31^) and has been manufactured into thin fibers to guide light, but has not been tested in brain in vivo with respect to biocompatibility of 126 ± 5 µm PDMS fibers and light transmission in size 71 ± 10 µm, as mono fibers (without cladding)^29,32–34^, (200 µm PDMS^29^; 100–130 µm polycarbonate^32^; 50–80 µm polycarbonate^33^; 250 µm PDMS^34^).

The aim was to clarify the potential of thin mono PDMS wave guides for long term optogenetic manipulation of neuronal activity, with special focus on the light transmission and biocompatibility aspects which is crucial for long term use. In the present study we used Sylgard 184 which is an easy accessible commercial PDMS often used for cell cultures and in pre-clinical implants, with no known toxic effects^35,36^. Due to its 2-component nature, the physical properties (Young’s modulus, refractive index etc.) can be tuned for individual needs. We also developed a pulling method to manufacture thin PDMS fibers.

We conclude that PDMS fibers have an improved biocompatibility compared to conventional glass fibers and enable in vivo excitation of channelrhodopsin (ChR2) infected CA3 neurons in rats.

## Results

### PDMS fiber production protocol

The new production setup for PDMS fibers facilitated production of optical fibers from Sylgard 184 in sizes from 20–200 µm. The semi-cured (9–10 h at 21–23 °C or 6–7 days at 4 °C (to get the desired viscosity)) Sylgard 184 (siloxane:curing agent = 3:1 (w/w)) was placed on a 3 mm wide metal rod with a cavity at the top. The metal rod was placed with the PDMS facing downwards on the linear actuator (ThorLabs, LTS150C). The “coupling” fiber (Thorlab, FT200UMT) was placed under the PDMS sample, and the PDMS sample was lowered to make contact and adhere to the “coupling” fiber. The PDMS fiber pulling process was monitored with a digital microscope (Media-Tech MT4096) and pulling speed (0.01–0.1 mm/s) and time (extra curing time is sometimes needed) were adjusted to get the desired fiber dimensions. The setup is graphical depictured in Supplementary Fig. 1.

### Young’s modulus

The fiber production method described in this paper gives optical fibers produced from Sylgrad 184 (3:1 w/w) a Young’s modulus of 0.31 ± 0.027 MPa. The applied force was linear for all 6 fibers in the tested elongation range (1–10 mm) with a mean R-squared of the linear regression of 0.996 ± 0.0011 (mean ± SD).

### Histological comparison of 125 µm glass and 126 ± 5 µm PDMS as optical waveguides

**Figure 1:**
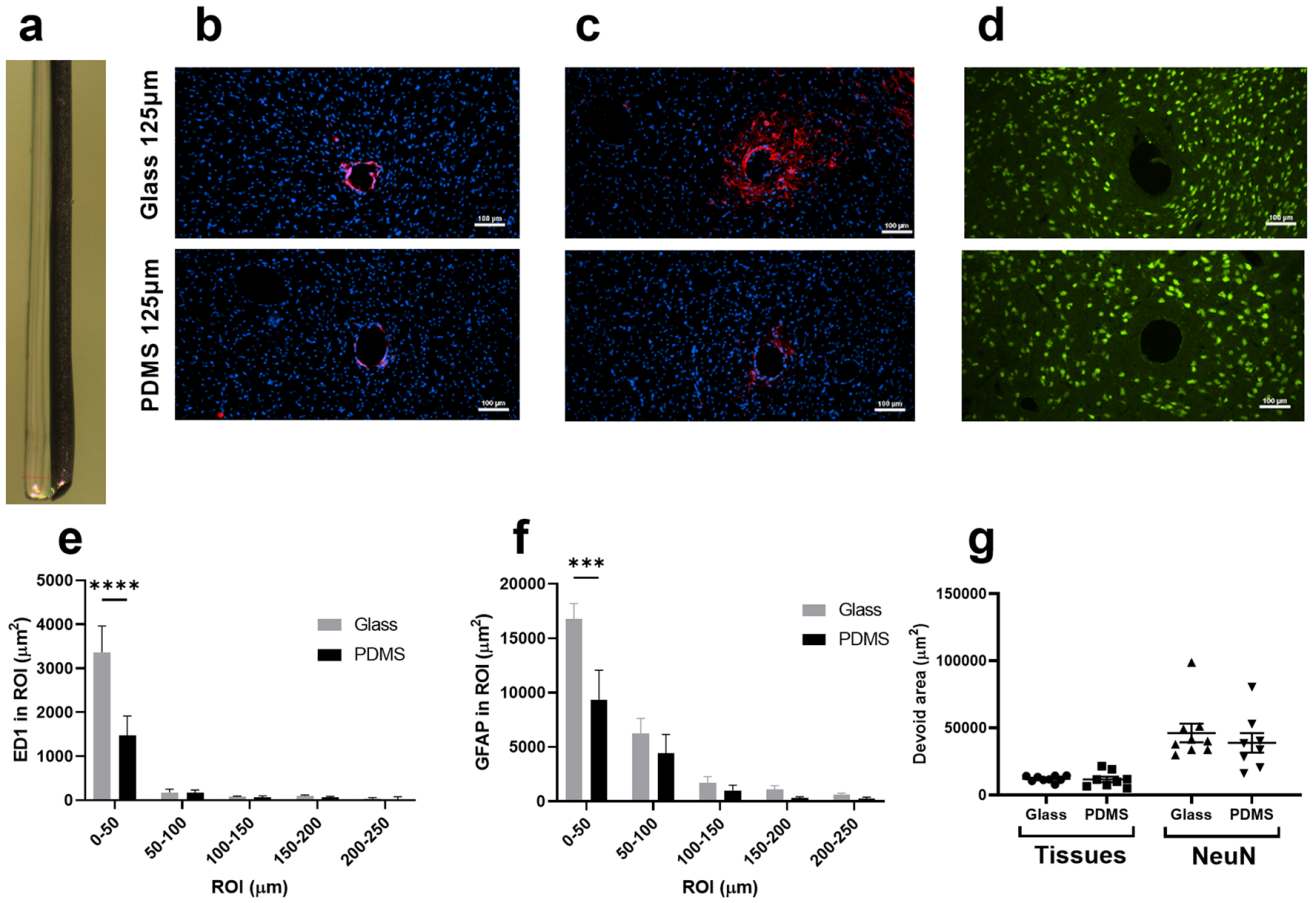
PDMS offers higher biocompatibility than similar size glass as light delivering device in vivo. Horizontal sections of a dorsoventral implantation of 126 ± 5 µm PDMS fibers and 125 µm glass fibers. (**a**) Representative picture of a PDMS fiber attached to a tungsten guide with gelatin. The total width of the construct in the picture is 238 µm. (**b**) Representative pictures of  and  stainings from (top) a glass fiber and (bottom) a PDMS fiber. (**c**) Representative pictures of  and  stainings from (top) a glass fiber and (bottom) a PDMS fiber. (**d**) Representative pictures of  stainings from (top) a glass fiber and (bottom) a PDMS fiber, the scale bars in (**a**–**c**) is 100 µm, all pictures are 10 × magnification. Each row is from same respective animal. (**e**) Quantification of area of ED1 activation, expressed as the computed pixel intensity above threshold in the respective ROIs (two-way repeated measures ANOVA, material × distance interaction, F(4,52) = 6.203, p = 0.0004) PDMS N = 7 and glass N = 8. (**f**) Quantification of area of GFAP activation, expressed as the computed pixel intensity above threshold in the respective ROIs (two-way repeated measures ANOVA, material × distance interaction, F(4,52) = 4.623, p = 0.0029). PDMS N = 7 and glass N = 8. (**g**) Graphic presentation of tissues area void and NeuN area void (tissues devoid, Mann–Whitney test p = 0.48; NeuN devoid, Mann–Whitney test p = 0.42) PDMS N = 8 and glass N = 9. All data is presented as mean ± SEM. Sidak post hoc analysis is represented by the stars (**d**–**f**) ***p < 0.001 and ****p < 0.0001.

Immunohistochemical evaluation of microglia and astrocytic activation of equal size implants was assessed by ED1 and GFAP stainings. Briefly, the ED1 and GFAP stained area was quantified by using a fixed ratio above the background as detection threshold and the stained area for each ROI was plotted. The ED1 staining gradually decreased in intensity with increasing distance from the implant in thalamus for both materials. Statistical analysis of ED1 staining in vicinity of the implant showed a significant interaction of fiber material and distance in thalamus (RM two-way ANOVA, F(4, 56) = 6.304, p = 0.0003) (Fig. 1b,e). The post hoc analysis revealed a significant lower ED1 staining around the PDMS fiber compared to glass in ROI 0–50 µm (p < 0.0001), corresponding to a 56% decreased microglia response.

The GFAP staining revealed an increased astrocyte activation close to the PDMS and glass implants of equivalent size. Statistical analysis of GFAP revealed a statistical interaction between fiber material and distance from fiber (RM two-way ANOVA, F(4, 53) = 4.623, p = 0.0029) (Fig. 1c,f). The post hoc analysis revealed a significantly lower GFAP staining around the PDMS fiber compared the glass in ROI 0–50 µm (p < 0.001), corresponding to a 44% decreased astrocytic activation response.

Neuronal (NeuN) and tissue voids were not different between PDMS optical fibers and glass fibers of equivalent diameter (125 µm) (Fig. 1d,g) (tissues void, Mann–Whitney test p = 0.99; NeuN void, Mann–Whitney test p = 0.38).

### PDMS fibers transmit sufficient light to activate channelrhodopsin in vivo

**Figure 2:**
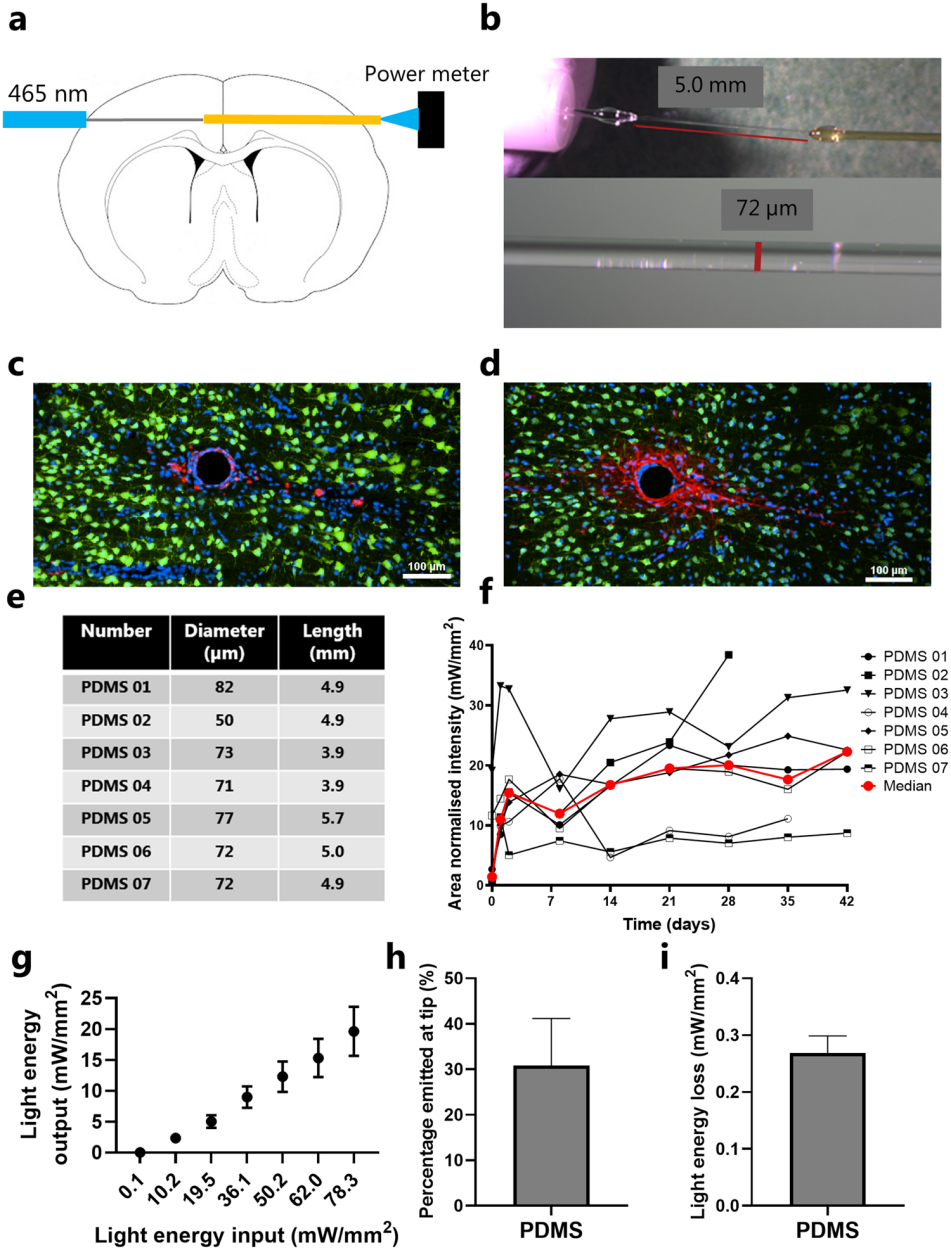
Thin PDMS fibers transmit high light intensities in vivo. (**a**) Schematic drawing of how the PDMS fiber was implanted. (**b**) A representative example of a PDMS optic fiber construction for measuring light transmission in vivo. The pictured waveguide is PDMS06. Top is 7 × and bottom is 80 ×. (**c**) Representative picture of ,  and  stainings from a PDMS fiber (10 ×) (PDMS 04). (**d**) Representative picture of ,  and  stainings from a PDMS fiber (10 ×) (PDMS 04). (**e**) Table with the PDMS fibers specifications. (**f**) Graphic presentation of the light flow in individual fibers over time at maximum LED output. The light power is normalized to the transection area of the PDMS fiber. Each data point is the average of three measurements. (**g**) Average light energy output at the PDMS fiber tip in relation to the maximum calculated light energy input (mean ± SEM). (**h**) Light emission at fiber tip as percentage of input (mean ± SEM). (**i**) Light energy emitted from the PDMS fiber shank (mean ± SEM). *p < 0.05. Scale bars in (**c**) and (**d**) are 100 µm.

In our effort to evaluate fibers made from a single layer of PDMS as a biocompatible alternative to glass optical fibres for optogenetic use, we tested 71 ± 10 µm PDMS fibers light transmission properties in vivo (length of 4.7 ± 0.64 mm (mean ± SD)) (see “Methods” and Fig. 2a,b). On average, the light transmission increased dramatically the first week, to reach a plateau. All fibres showed a good performance and 5 of 7 showed light transmission above 10 mW/mm^2^, whereas 2 of 7 had intensities between 5 and 10 mW/mm^2^, which would be sufficient to excite nearby opsin positive neurons in a theoretical distance of > 500 µm from the light delivering tip^37,38^. Data from individual animals and median are summarised in Fig. 2e,f. The maximum light output from the PDMS fiber, when assuming 100% internal reflexion in the PDMS, was calculated as the output of the Plexon fiber stubs in relation to the transection area ratio of the PDMS fiber (Ø 71 ± 10 µm) and the Plexon fiber stub (Ø 200 µm) (Fig. 2e,f). The loss of light along the PDMS fiber was calculated as ratio of light input compared to the light output at the fiber tip relative to the fiber surface area. 31 ± 10% of the input light (78 ± 7.5 mW/mm^2^) was emitted from the PDMS fiber tip (Fig. 2h). The light emitted along the fiber was calculated to 0.27 ± 0.03 mW/mm^2^, assuming a uniform emission along the fiber (Fig. 2i).

### Acute electrophysiological evaluation of PDMS as light delivering device in vivo

**Figure 3:**
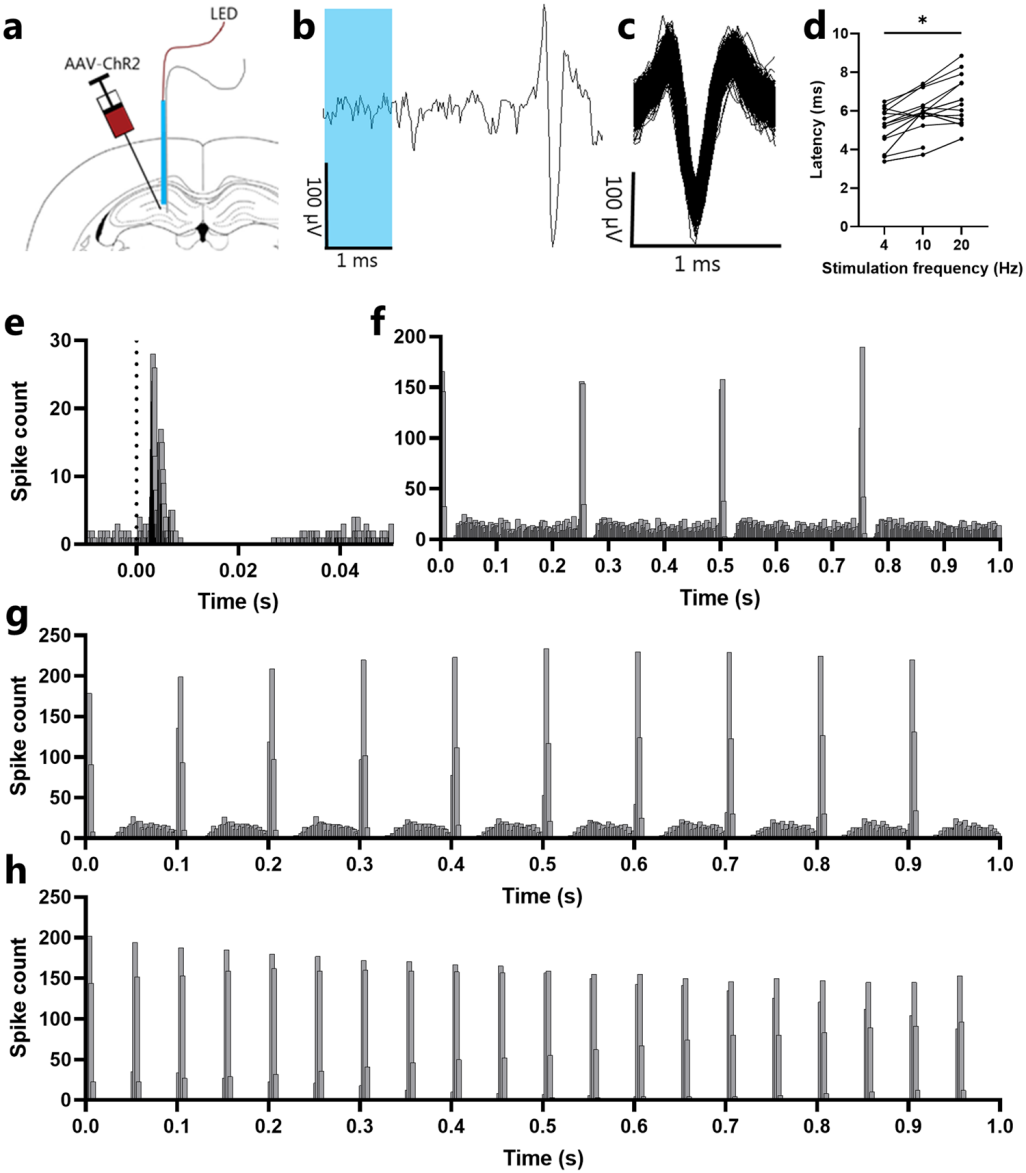
Acute in vivo single unit recordings of ChR2 transfected CA3 neurons. (**a**) Schematic drawing of the experimental setup. (**b**) Extracellular recorded action potential with a 1 ms light pulse. (**c**) Waveform overlay of 1400 waveforms after spike sorting. (**d**) Graphic presentation of the median latency of each recorded neuron. The median latency of the evoked action potentials increase with increasing stimulation frequency. (**e**) 50 ms graphical representation of peristimulus time histogram of optogenetic stimulation at 4 Hz, peak at 3.2 ms and median latency at 3.38 ms (2_3573). Bar width is 0.1 ms. Dotted line represents a 1 ms light pulse. (**f**–**h**) 1 s graphical representation of peristimulus time histogram of optogenetic stimulation at (**f**) 4 Hz, (**g**) 10 Hz and (**h**) 20 Hz, recording 2_3573. Bar width is 2 ms. *p < 0.05.

To further evaluate the light transmission through implanted PDMS mono fibers we recorded hippocampal CA3 neurons extracellularly 3–4 weeks after they were injected with AAV2/1-CaMKIIα-ChR2(H134R)-mCherry. We successfully recorded action potentials in 16 neurons in hippocampal CA3 elicited by light pulses with a median onset delay of less than 7 ms at 4 Hz stimulation (rats = 4, neurons = 16) (Fig. 3 and Table 1). In general, the onset latency of the evoked action potentials increased with an increased pulse frequency. The mean of the median latency was significantly different between the 3 stimulation frequencies (Kruskal–Wallis test, p = 0.0097). The pairwise comparison revealed a significant increase of the median latency between the LED pulse and the evoked action potential when stimulation frequency was increased from 4 to 20 Hz (Dunn’s pairwise comparison, p = 0.011) (Fig. 3d). Our results thus show that the customised optical PDMS fiber is able to drive the generation of action potentials in single neurons by activation of ChR2-driven depolarisation in the recorded neuron.

**Table 1:** All recorded neurons responding to light with a median latency below 7 ms. The table show the median, 25% and 75% percentile of the latency in seconds.

Rat/cell#		4 Hz	10 Hz	20 Hz	Rat/cell#		4 Hz	10 Hz	20 Hz
1_1	25% percentile	0.00358	0.00448	0.00575	3_1	25% percentile	0.00520	0.00462	0.00473
	Median	0.00370	0.00598	0.00658		Median	0.00588	0.00571	0.00578
	75% percentile	0.00393	0.00946	0.01235		75% percentile	0.0453	0.00648	0.00693
1_2	25% percentile	0.00318	0.00335	0.00395	3_2	25% percentile	0.00318	0.00380	NA
	Median	0.00338	0.00373	0.00455		Median	0.00363	0.00410	NA
	75% percentile	0.00353	0.00403	0.00550		75% percentile	0.00413	0.00473	NA
1_3	25% percentile	0.00479	0.00330	0.00421	3_3	25% percentile	0.00536	0.00600	0.00685
	Median	0.00533	0.00593	0.00555		Median	0.00560	0.00628	0.00743
	75% percentile	0.00591	0.00660	0.00689		75% percentile	0.00598	0.00655	0.00793
1_4	25% percentile	0.00426	0.00465	0.00423	4_1	25% percentile	0.00555	0.00668	0.00728
	Median	0.00465	0.00590	0.00528		Median	0.00593	0.00730	0.00829
	75% percentile	0.00989	0.01263	0.00810		75% percentile	0.00648	0.00790	0.00904
2_1	25% percentile	0.00500	0.00559	0.00679	4_2	25% percentile	0.00319	0.00595	0.00708
	Median	0.00518	0.00588	0.00745		Median	0.00648	0.00723	0.00790
	75% percentile	0.00530	0.00608	0.00798		75% percentile	0.00746	0.00861	0.00933
2_2	25% percentile	NA	0.00398	NA	4_3	25% percentile	0.00418	0.00560	0.00465
	Median	NA	0.00423	NA		Median	0.00531	0.00618	0.00603
	75% percentile	NA	0.00460	NA		75% percentile	0.00692	0.00770	0.00928
2_3	25% percentile	0.00448	0.00452	0.00493	4_4	25% percentile	0.005869	0.006719	0.008150
	Median	0.00453	0.00524	0.00538		Median	0.006250	0.007400	0.008850
	75% percentile	0.00465	0.00579	0.00604		75% percentile	0.006625	0.007900	0.009425
2_4	25% percentile	0.004263	0.004650	0.004225	4_5	25% percentile	0.005400	0.003306	0.004675
	Median	0.004650	0.005900	0.005275		Median	0.006125	0.005650	0.006325
	75% percentile	0.009888	0.01263	0.008100		75% percentile	0.006725	0.006344	0.007000

## Discussion

In this study, we established and tested the functionality of micro PDMS mono-fibers for optogenetic activation of ChR2 in vivo. Sylgard 184 offers a board spectrum of opportunities for customising optic fibers within preclinical research. The possibility to produce thinner, more flexible fibers and close to brain density is here shown to provoke a significantly decreased tissue reaction compared to that of glass fibers of same diameter. The improved biocompatibility and thus degree of preservation of physiological conditions in the tissue is of interest both in optogenetic studies and when optogenetic techniques are combined with electrophysiological recordings. Along with increased biocompatibility, the mono-fiber concept also offers a simplified and fast production method compared to a traditional bilayer construct.

An important result of the present study is that a monolayer PDMS optical fibers with a diameter as small as 71 ± 10 μm are able to deliver enough light to directly evoke action potentials in electrophysiologically recorded neurons with virial expression of ChR2(H134R). The median latency of evoked action potentials was as low as 3.38 ms at 4 Hz. The short latency of the 16 recorded neurons suggest that the light directly (in most cases) drives the generation of action potentials of the recorded neurons.

Considering the kinetics of ChR2(H134R), the average opening time of the ChR2(H134R) channel variant is approximately 2.5 ms which means that the latencies for neuronal activation found are compatible with direct activation. As the LED power and pulse wide were adjusted, to the individual neurons, to evoke spike activity with a high probability with the lowest possible light energy output. We did not observe any photoelectric artefacts and by adjusting the light power to the individual neurons the effect on the local field potential was minimised. We cannot rule out bi- or trisynaptic activation of recorded neurons at latencies > 5–6 ms^39^. The activation time of ChR2(H134R) in vivo might be increased slightly by low transfection or longer distance between the stimulated neuron and optical fiber. A longer distance in the tissues will cause increased light absorption and scattering which together will cause less light energy to reach the ChR2(H134R) channels and therefore a slower depolarisation to the threshold^38,40^. Also our use of low light intensity to minimize local field potential deflection, will cause a slower depolarization, causing a longer latency. Our data clearly show that micro PDMS fibers are capable to synchronize the firing of presumed CA3 neurons in the frequency range of 4–20 Hz (Fig. 3e–g). By increasing the stimulation frequency of the LED we found an increase in the latency of the evoked action potentials which may be related to the relative slow off-kinetics of the ChR2(H134R) opsin variant (25–30 ms) causing a longer repolarization time thereby increasing the relative refractory period at high frequencies^40^. However, we cannot exclude that intrinsic properties of CA3 neurons are not involved^41^. A similar phenomena has been observe previously in optogenetic experiments^42^.

Another important finding in our study is the observed decrease in microglia and astrocytic activation around both small and size-matched PDMS fibers as compared to glass. This improvement in biocompatibility gives the opportunity to study the signalling in more intact circuitries close to the probe implantation. A similar finding was also reported by Cao et al*.*^29^, using larger fibers (200 µm diameter) and a bilayer construct. In our study, we tested PDMS fibers of both 71 ± 10 µm and 126 ± 5 µm diameter against 125 µm glass fibers. The experiments using 71 ± 10 µm and 126 ± 5 µm PDMS fibers, cannot be directly compared as the surgical methods and implanted brain areas were very different. However, they can be compared to their respective controls, and by using a smaller size PDMS fiber (71 ± 10 µm), reduction in GFAP activation was greater than for 125 µm PDMS fibers. The surface of an implant may play a role of any device in the organism’s immune reaction directed towards a foreign body. The surface properties and chemistry of Sylgard 184 and glass are very different being hydrophobic and hydrophilic, respectively, and might also contribute to the differences in biocompatibility of Sylgard 184 and glass fibers. The microglia and astrocytic responses was quantified 6 weeks after the implantation to evaluate the impact on the chronic inflammatory response to the implants. In support, at 2–6 weeks the light transmission (Fig. 2f) was more stable which might indicate that the inflammatory response is in a chronic and slow developing phase.

Density and flexibility are important parameter in striving for better biocompatibility. It has been shown that implants with lower density show less activation of microglia and astrocytes compared to high density implants of same size, shape and surface properties^11^. The density of glass is on average 2.2 g/cm^3^ whereas Sylgard 184 has a density of 1.03 g/cm^3^.

Flexibility to absorb and follow micro motions from the brain is pivotal for the stability and stationarity of an optic fiber or electrode in the brain tissues^13^. It is shown that a reduction in Young’s modulus increases biocompatibility and in comparison; PDMS is much softer (Young’s modulus 50,000 times lower) then glass^43–48^. The low density and low Young’s modules (PDMS: D = 1.03 g/cm^3^ and E = 0.31 ± 0.027 MPa) is likely the main reason for our results showing a low microglia and astrocytic reactivity of size-matched and small PDMS implants compared to implanted glass fibers. Another major factor affecting biocompatibility is the size of the probe^10^. The ability to produce small size PDMS fibers is thus an additional advantage compared to most multimodal optic fibers in glass with the smallest prefabricated diameter being 125 μm (cladding included).

The flexibility of Sylgard 184 depends on the curing process and Sylgard 184 cured at 22–25 °C has an Young’s modulus of 0.31 ± 0.027 MPa which is approximately 250 times more than brain tissues, but 225,000 times small than a pure glass fiber (E = 70 GPa)^49^. Young’s modulus of Sylgard 184 is tuneable and proportional to the curing temperature and high curing temperature gives a higher Young’s modulus. In this way, we can aim at lowering Young’s modulus by slowly curing the PDMS fibers at room temperature (at 25 °C the curing time is 48 h)^27,50,51^. Very thin glass fibers are extremely fragile without the protecting outer plastic coating, even at 125 μm diameter glass^52^. On the other hand, small diameter PDMS fibers are extremely resilient and tolerates 100% length extension, compression and bending (> 360°)^53^.

The curing process of Sylgard 184 starts when the curing agent is added. With our approach it is possible to pull a fiber of any wanted diameter and length from partially cured Sylgard 184. When fully cured our in vivo tests revealed a light transmission of 5–40 mW/mm^2^. To our knowledge, the transmission of light through PDMS fibers in vivo has not been tested previously. The measured light density (Fig. 2f) shows fluctuations in the output between different time points. It is possible that the fluctuations reflects different compositions of microglia adhering to the fiber at different time points. The major fluctuations are seen at time 1–14 days, being more stable after day 14–28 which might relate to that immune responses reach a more chronic and stable state after day 14–28^48,54,55^.

The activation power density of cation channel channelrhodopsin is 0.5–1 mW/mm^2^ of 470 nm light. Point mutations of channelrhodopsin has lowered the light density need for activation and red shifted variants like Chronos and Chrimson are as low as 0.1 and 0.015 mW/mm^2^, respectively^40^. Scattering and absorption of light are the most important factors determining the activation distance in optogenetic experiments. The tissue absorption of light is highest at high frequency light and decreasing at lower frequencies. For this reason red and near infrared light is more favourable, but the light sources are usually less powerful then blue LEDs and lasers. For 465 nm light only 25% of the light density will reach as far as 250 μm and less than 10% will reach further than 1000 μm^37,38^. Under the in vivo conditions and in the perspective of blue light scattering and absorption, the theoretical activation distance of ChR2 with our PDMS fibers with a diameter of 71 ± 10 µm is more than 500 µm. In general, hyperpolarizing opsins (H^+^- and chloride-pumps) need a higher light density to be activated ranging from 1.9 to 8 mW/mm^2^ (EDP50) while the new bidirectional opsin BiPOLES are much more sensitive to light (0.1 mW/mm^2^)^56^ (ChR2; 0.5–1 mW/mm^2^, Chronos; 0.1 mW/mm^2^ and Chrimson; 0.015 mW/mm^2^)^40^. Therefore, dependent on the output power of the light source the light intensity levels for activating hyperpolarising opsins are also within the possible output range of PDMS optical fibers.

Micro LEDs are a developing technology and has the advantage over optical fibers like high power and that the light source is mounted directly in the tissue where light emission is desired. This gives a very precise control over the light emission, as there is no coupling loss. However, the need for a rigid backbone of e.g. silicon (E = 150 GPa) is a major disadvantage for micro LEDs, and flexible backbones are not yet standard in implants for deep targets^57^. Furthermore, heating of the tissues is also a relevant consideration that can change neural firing when using both micro LEDs and lasers^58^*.* This together with a very advanced production line, risk of shorts and breaks are a dominant source of failure of micro LEDs^59^. Powering of the LED might also introduce noise if used together with electrophysiological recordings.

A disadvantage of PDMS as the only material is the increased risk for adhesion of small dust particles to the surface of PDMS which, unless prevented by using clean room facilities, can reduce the fraction of total internal reflected light. Compared to glass the refractive index of in vivo implanted Sylgard 184 may change over time, as it can absorb small amounts of water. The refractive index over time of implanted PDMS fibers was not studied, but the stabilized light transmission may indicate that the refractive index is relative constant after 14–28 days. The soft and flexible nature of PDMS also means that it is necessary to use a guide pin when implanting. In the presented data we used a 100 µm tungsten guide pin which increases the overall stab injury of the implanted device. The immune response to the stab injury of the guide pin on its own was not investigated, but previous published data shows that a gelatinised guide pin leaves almost no detectable impact after 6 weeks^60^. Another drawback with of a single layer optical fiber of PDMS is the relative low refractive index (Sylgard 184 10:1 mixture RI: 1.425 (RI: refractory index)^26^) in relation to brain tissues and CSF (RI = 1.39–1.41 and RI = 1.33, respectively)^61^. The small difference in refractive index between Sylgard 184 and brain tissues in the single layer construction cause leakage of light throughout the length of the fiber. The leakage of light along the fiber could potentially activate or inhibit neuron all along the fiber. RI of Sylgrad 184 is slightly lower for light with longer wave length. However, the difference in RI between Sylgard 184 and brain tissue still supports total internal reflection (RI: 1.4483 @ 405 nm, 1.4348 @ 532 nm and 1.4295 @ 635 nm, 10:1 mixing ratio)^62^. Our calculations show that the light emission along the surface of the fiber, assuming a uniform emission and not considering the loss at the PDMS-glass fiber coupling, is 0.26 ± 0.08 mW/mm^2^ (mean ± SD) which is not sufficient to activate the most common variants of ChR2 (Fig. 2). Furthermore, minimum 31 ± 10% (mean ± SEM) of the light transmitted into the fiber was emitted from the tip. This percentage is probably an underestimation, as some light is most likely lost in the connection between the PDMS fiber and the optical fiber. However, in models with regional expression after viral injections or for optogenetic phenotyping of electrophysiologically recorded neurons, this technology offers an outstanding opportunity for custom made highly biocompatible optical fibers for preclinical neuroscience^41,63^. One option to accommodate the lower difference in RI of Sylgard 184 and brain tissues is to increase the RI of the Sylgard 184 by adding a higher ratio of curing agent. Another possibility is optimize the curing temperature. By using a 1:1 ratio and a curing temperature of 240 °C a fiber will reach a RI as high as 1.45^26,27^. This would, in theory, improve transmission in in vivo applications. An alternative to the single layer construction is a traditional bilayer construction for instance coating with another PDMS with a lower RI or a Teflon coating.

## Conclusion

We have provided evidence for the advantage and function of PDMS as optical fiber in optogenetics of 71 ± 10 µm diameter. Our data indicates that PDMS fibers are more biocompatible than commonly used optical glass fibers. Light transmitted through PDMS reach sufficient intensity to evoke action potentials with a short onset latency in vivo. A further advantage is that PDMS fibers can easily be pulled into almost any wanted diameter and length.

## Methods

### Animals

Female Sprague Dawley rats from Taconic (Denmark) (N = 20) weighting between 250–350 g were used for all in vivo experiment. Animals received water and food ad libitum and were housed in a 12 h day night cycle (dark 10 am–10 pm). Room temperature and relative humidity were kept constant at 21 °C and 65%. The experiments follow the ARRIVE guidelines and were performed in accordance with regulations and legislation stated by the European Union and the law on animal welfare in Sweden. All animal experiments were preapproved by Malmö/Lund Animal Ethics Committee on Animal Experiments (registration number R4689-19), regulated by the code of regulations of the Swedish Board of Agriculture. These regulations, including directives from the European Union, follow the law on animal welfare legislated by the Swedish parliament. Final sample size was based on power analysis of pilot experiments.

### Fabrication of PDMS fibers

Sylgard 184 was mixed in a 3:1 weight ratio of silicone elastomer base and curing agent, respectively. The mixture was left to cure slowly at 4 °C for 6 days to reach a viscosity and strength at which it was possible to pull thin fibers at room temperature. The fibers were pulled vertically using a micromanipulator at a speed of 0.01–0.1 mm/s. This setup allowed us to fabricate PDMS fibers of 20–200 µm in diameter and up to 7 cm in length. When the fiber was pulled to the desired diameter it was left to cur for 24 h before demounting. To complete the curing process the fibers were afterwards cured at 70 °C for 2 h.

PDMS fiber of 126 ± 5 µm (118–135 µm) was fabricated as described above and attached to a straight tungsten wire (100 µm in diameter) with 5% 289 bloom strength gelatin (Gelati MedellaPro 1500) which allowed implantation of the soft PDMS fiber. The gelatinised fiber/wire construction was placed 2 h in 100% relative humidity chamber at room temperature. Followed by 2 h in 85% relative humidity at room temperature and finally in a dry chamber (10–25% relative humidity at room temperature) until usages. The slow drying sequence was used to prevent asymmetrical drying which can cause the probe to bend during drying. A 125 µm optic fiber in glass (Thorlabs, FG105UCA, 105 µm core) was attached to a 100 µm tungsten wire similar to the PDMS fiber, for direct histological comparison. The overall dimensions of the PDMS-tungsten and glass-tungsten constructs were 226 ± 5 × 126 ± 5 µm^2^ and 225 × 125 µm^2^, respectively.

### Young’s modulus

Young’s modulus (E) was calculated based on the formula for stiffness [E1] and [E2]. Stiffness k was found by measuring the applied force to give a specific change in length δ. The PDMS fiber was attached to a small weight and placed on a scale (0.1 mg resolution) in one end and a digital Vernier height gauge (0.01 mm resolution) in the other. The force applied to give a change in length was calculated as the change in weight. Each fiber (N = 6) had 10 measurements, from 1 to 10 mm extension. The measured fibers had a diameter of 162 ± 13 µm and a length of 24 ± 4 mm. The applied force was plotted as a function of the change in length, stiffness k was found by linear regression and inserted in the equation for E [E2].

E1 $$k = \frac{F}{\delta } = \frac{E \times L}{A}$$

E2 $$E = \frac{k \times A}{L}$$

### Implantation of fibers and electrodes

Surgical anaesthesia was induced with 5% isoflurane in a 50:50 mixture of O_2_ and N_2_O. After induction and mounting in the stereotaxic frame (Neurostar^®^, Tübingen, Germany) the isoflurane were lowered to a level of surgical anaesthesia (typically 1.2–2.5%). The eyes were kept moist by ophthalmic ointment of Sodium hyaluronate 2.0 mg/ml (ZilkEye, Bohus BioTech AB, Strömstad, Sweden) and body temperature was kept constant at 37 °C. After removing the hair and disinfecting the skin with 0.1% chlorhexidine the midline was infiltrated with a lidocaine (20 mg/ml) injection. An incision exposed the skull providing access to measure bregma, lambda and head tilt. A craniotomy were made right above both thalami (AP: − 3.20, ML: ± 2.2)^64^. Dura mater was carefully incised to reduce friction during subsequent insertion of the optical probes. The PDMS and tungsten construct was lowered at a speed of 1 mm/s to a depth of 6 mm below the brain surface to reach thalamus (Fig. 1a). The PDMS or glass fiber was detached from the tungsten wire by spraying 37 °C isotonic saline on the part of the construct protruding out of the brain. After 10 min the tungsten guide wire was slowly explanted from the brain (0.1 mm/s) leaving the PDMS or glass fiber inside the brain. Finally, the PDMS fiber was secured with dental cement (Tetric evoflow A1, Ivoclar Vivadent). Contralateral to the implantation of the PDMS fibers, the glass and tungsten construct was implanted and secured to the skull with dental cement. The skin was closed with sutures and the rat was given 0.05 mg/kg buprenorphine.

### Implantation and in vivo measurements of PDMS light transmission

In order to measure the light transmission in PDMS fibers in vivo we mounted the PDMS waveguide serially between two ordinary optical waveguides and implanted the assembly horizontally through the brain (Fig. 2a). The connection to the LED light source (PlexBright table-top LED module 465 nm, 34.9 mW output, Plexon) was established through a Plexon 200/230 µm ferrule (2 mm), the PDMS fiber was attached using Sylgard 184 3:1 mix ratio (w/w). The other end of the PDMS fiber was attached to an optical fiber with a core diameter of 200 µm (Edmund optics, #57-068). This connection was also secured with Sylgard 184. The PDMS part of these assemblies had a diameter of 71 ± 10 µm (mean ± SD, 50–82 µm) and a length of 4.75 ± 0.64 mm (mean ± SD, 3.9–5.7 mm) (Fig. 2b,c).

General surgical procedures were the same as described above in section “Implantation of fibers and electrodes”. A rostrocaudal and a mediolateral incision (at the level of bregma) were made and the bone was thoroughly cleaned. At the rostrocaudal level of bregma a hole was drilled in the skull (AP 0.0 mm, ML ~ 6.00 mm and DV − 2.00 mm, in relation to bregma). This procedure was repeated at the contralateral side. The waveguide assembly (Fig. 2a) was implanted horizontally using a custom made holder. The assembly was implanted to a depth where the tip of the Plexon ferrule was just above the brain surface. Three stainless steel anchoring screws were attached to os paritalis and os frontalis and the implant was secured with dental cement (RelyX™ Unicem, 3M ESPE). The fiber protruding at the contralateral side was cut to a desirable length and polished. This setup allowed us to measure transmission of light through the PDMS mono fiber in the brain in vivo. After finished implantation the skin was closed using sutures and the rat received 10 ml/kg 0.9% NaCl SC and buprenorphine SC (0.05 mg/kg).

The light emission intensity was measured at day 0, 1, 2, 8, 14, 21, 28, 35 and 42. The rat was briefly anaesthetised using 2% isoflurane in a 50:50 mix of O_2_ and N_2_O (v/v). The Plexon ferrule was connected to a LED (Plexon table-top LED module (465 nm) and Plexon high performance optical cable). A photodetector was placed contralateral to measure exiting light emission (Thorlabs, PM100D and S120C). The light emission was measured at 7 different input intensities corresponding to a LED current input of 0, 25, 50, 100, 150, 200 and 300 mA (these current LED inputs are equivalent to a light power output of 0, 13, 26, 47, 66, 81 and 103 mW/mm^2^ at the tip of the optic fiber) and repeated 3 times per time point. The emission intensity was measured and normalised to the transection area of the PDMS fiber (Fig. 2g). Two rats lost the ferrule at day 28 and 35 but data points were still included until the day the implant was lost.

### Viral injection and acute extracellular recordings

General surgical procedures were the same as described above in section “Implantation of fibers and electrodes”. A craniotomy were made right above both hippocampi (AP: − 3.60, ML: ± 2.25)^64^. An ultrafine borosilicate capillary pulled under heat and broken to ~ 50–70 µm were lowered slowly to the injection site (50 µm/s, DV: − 3.00)^64^. 100 nl of 1.2 × 10^12^ GC/ml AAV2/1-CaMKIIα-ChR2(H134R)-mCherry was injected at 20 nl/min rate. The capillary was left in place for 5 min to reduce backflow. After injection the capillary was retracted, the skin was sutured and the rat was administrated buprenorphine SC (0.05 mg/kg).

3–4 weeks after the viral injection the animals was subjected to acute extracellular recordings (Fig. 3a). The electrode, a 50 µm tungsten wire coated with PTFE (Advent Research Materials Ltd, Cat. No. W5620) was attached to a PDMS fiber (82 µm) with LS6941 PDMS (1:3 mix ratio w/w). The tungsten wire protruded 250–500 µm below the distal end of the PDMS waveguide and 50 µm was de-insulated with low power UV laser irradiation. The anaesthesia and implantations were performed as described in above section. The optrode was slowly lowered to a start position of 800–1000 µm above the area of interest. The signal was amplified 250 ×, high band filtered (300–5000 Hz) and continuously monitored on an oscilloscope and auditive (OmniPlex^®^ Neural Recording Data Acquisition System, Plexon Inc., USA). The LED was switched on (4 Hz and 5 ms puls-width) and the optrode was lowered at a speed of 2 µm/s. The power (calculated; 2–17 mW/mm^2^) and pulse wide (1–10 ms, square pulse) of the LED were adjusted to the lowest value possible still evoking action potentials. At the end of the recording session the rat was perfused under deep anaesthesia with app. 100 ml saline followed by post fixation with 4% PFA. The recording sites were then retrospectively reconstructed.

### Perfusion and tissues processing

Histological evaluation was performed 6 weeks after the implantation. Before transcardial perfusion the rats were anaesthetised with pentobarbital i.p. (dosed until effect, which was evaluated as absence of the deep reflex, typically 90–180 mg/kg). The left ventricle was open and an 18 gauge blunt needle was inserted. The circulatory system was perfused with isotonic NaCl for 5 min (app. 100 ml), followed by 20 min of ice cold 4% paraformaldehyde (pH 7.4) (app. 350 ml) and finally perfused with isotonic NaCl for 5 min (app. 50 ml). For the experiments using 71 ± 10 µm PDMS fibers to measure the light transmission, the implant was carefully removed using a circular diamond saw so the PDMS part of the optical waveguide was kept inside the brain. For the size-matched comparison of 125 µm glass and 126 ± 5 µm PDMS fibers both fibers were removed from the brain. Immediately after removal the brain was transferred to at cryoprotective solution (20% sucrose in PBS solution (w/v)). Because the implants were removed from the brain before sucrose treatment the remaining voids shrunk in some brains. For the comparison of 125 µm glass and 126 ± 5 µm PDMS fibers both fibers were removed from the brain. The sucrose solution was changed 4 times with 12 h interval. After 48 h the brain was snap frozen in < − 60 °C isopentane, and stored at − 80 °C until further processing.

### Immunohistochemistry

The brains were sectioned on a cryostat (16 µm thickness) and mounted on Super Frost 1 plus slides (Mänzel-Gläser, Germany). The sections were washed for 10 min 3 times in PBS before the sections were blocked with goat serum and TritonX (60 min). The sections were incubated with primary antibodies in block buffer at room temperature overnight (NeuN, #104225 (1:500), Abcam, USA, and ED1, #MCA341R (1:250), AbD Serotec, UK or GFAP (1:500)). At day 2, the sections were first washed three times in PBS (3 × 10 min), followed by 2 h incubation with secondary antibodies and DAPI suspended in blocking buffer (DAPI, Invitrogen, USA (1:1000); goat anti-rabbit Alexa 594, Invitrogen, USA (1∶500) and goat anti-mouse Alexa 488, Invitrogen, USA (1∶500)). Before coverslips were mounted with DABCO (Sigma–Aldrich, Sweden), the sections were washed 3 times in PBS (3 × 10 min). Slides were examined and light microscopy images were captured using a DS-Ri1 digital camera (Nikon Instruments, Japan) mounted on a Nikon eclipse 80i microscope. The image acquisition and analysis were done using NIS-Elements BR software 3.05 (NIS-Elements, Nikon Instruments, Japan). The implanted areas were photographed using 10 × objective with the same gain, contrast and exposure times for the respective markers. The detection thresholds for GFAP and ED1 were set at a fixed ratio above the background intensity and applied to all acquired images. The detection threshold was found by averaging a manual set threshold for one image from each animal. Briefly, GFAP and ED1 staining were evaluated in 5 regions of interest (ROI) surrounding the implant: 0–50 µm (where zero corresponds to the border of the tissues void zone), 50–100 µm, 100–150 µm, 150–200 µm and 200–250 µm. The GFAP and ED1 stained area of each ROI were plotted for the PDMS and glass fiber implants and statically compared. For the statistical comparison each data point is an average of two horizontal brain sections from thalamus separated by minimum 96 µm, the area surrounding the implant was divided into ROIs, areas extending into the lateral ventricles were excluded.

The area devoid of detectable NeuN staining (NeuN devoid) was encircled and measured around the implants. The extent of the area deprived of tissues staining (tissues devoid) was also outlined and measured. NeuN devoid and tissues devoid encircled areas were analysed using NIS-Elements.

### Statistical analysis and signal processing

The GFAP and ED1 stained area surrounding the PDMS or glass fiber was divided into ROIs extending from the border of the implanted fiber with each ROI increasing the radius with 50 or 100 µm. The GFAP and ED1 stained area of each ROI for the PDMS and glass fiber implants were compared using a repeated measurement (RM) two-way ANOVA with Sidak post hoc analysis. The tissues and NeuN void areas were compared using Mann–Whitney test. All statistical analysis was performed using GraphPad Prims 9.4.0 software (Graphpad Software Inc., USA).

The latency between LED pulse and first action potential was analysed using in-built functions in Spike2 version 7.20 (CED, Cambridge, UK). Before analysis, spike trains were inspected to ensure that the train only contained spikes from a single neuron and that all spikes within a given time period were included in the analysis. A minimum of 100 stimulations were used for the analysis of single neuronal responses. Spike sorting was done using principle component analysis and the spike waveform overdraw function in Spike2. Interspike interval histograms were constructed for visual evaluation of refractory period violations. All included neurons had less than 1% refractory period violations (interspike intervals < 2 ms). The median latency of the different applied LED frequencies was compared using Kruskal–Wallis test followed by Dunn’s pairwise comparison.

### Supplementary Information

Supplementary Figure S1.

Supplementary Information.

## Data Availability

Data will be available on request by directly contacting corresponding author at mian@sund.ku.dk.
