# Supplementary figures and images for: Polydimethylsiloxane as a more biocompatible alternative to glass in optogenetics
Source: Sci Rep. 2023 Sep 26;13:16090. doi: 10.1038/s41598-023-43297-2 (PMC10522705; doi:10.1038/s41598-023-43297-2)

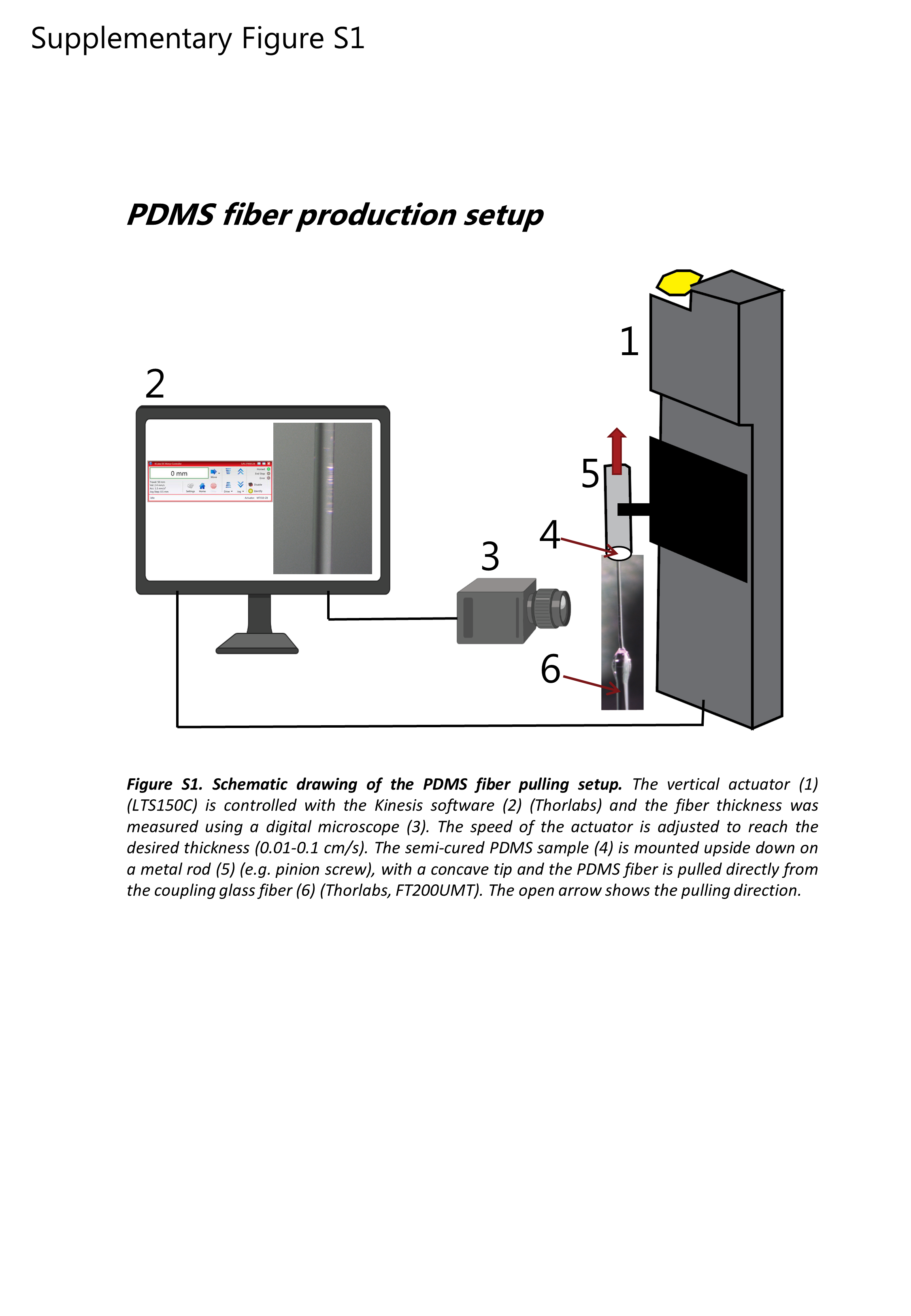

Supplement: Supplementary file 1 — Supplementary Figure S1. [file 41598_2023_43297_MOESM1_ESM.tiff]
