# Supplementary material for: Polydimethylsiloxane as a more biocompatible alternative to glass in optogenetics
Source: Sci Rep. 2023 Sep 26;13:16090. doi: 10.1038/s41598-023-43297-2 (PMC10522705; doi:10.1038/s41598-023-43297-2)
Supplement: Supplementary file 2 — Supplementary Information. [file 41598_2023_43297_MOESM2_ESM.docx]

***PDMS fiber production setup***

***Figure S1. Schematic drawing of the PDMS fiber pulling setup.*** *The vertical actuator (1) (LTS150C) is controlled with the Kinesis software (2) (Thorlabs) and the fiber thickness was measured using a digital microscope (3). The speed of the actuator is adjusted to reach the desired thickness (0.01-0.1 cm/s). The semi-cured PDMS sample (4) is mounted upside down on a metal rod (5) (e.g. pinion screw), with a concave tip and the PDMS fiber is pulled directly from the coupling glass fiber (6) (Thorlabs, FT200UMT). The open arrow shows the pulling direction.*
